# N-Myc-Interacting Protein Negatively Regulates TNF-α-Induced NF-κB Transcriptional Activity by Sequestering NF-κB/p65 in the Cytoplasm

**DOI:** 10.1038/s41598-017-15074-5

**Published:** 2017-11-06

**Authors:** Jingjing Hou, Shihao Jiang, Jiabao Zhao, Dong Zhu, Xinmeng Zhao, Jian-chun Cai, Si Qing Zhang

**Affiliations:** 1 State Key Laboratory of Cellular Stress Biology and School of Life Sciences, Xiamen University, Xiamen, Fujian, 361102 China; 2 Institute of Gastrointestinal Oncology, Medical College of Xiamen University, Xiamen, Fujian, 361004 China; 3 Department of Gastrointestinal Surgery, Zhongshan Hospital of Xiamen University, Xiamen, Fujian, 361004 China

## Abstract

NF-κB is a major regulator of gene transcription involved in immune, inflammation, apoptosis and stress responses. However, the regulation of NF-κB is not completely understood. Here, we report that the N-Myc and STATs Interactor (NMI), an IFN-inducible protein, is an important negative regulator of NF-κB activity. We found that NMI negatively regulates TNF-α-induced IL-6 and IL-1β production in HeLa cells. Overexpression of NMI inhibits NF-κB transcriptional activity, in contrast, depletion of NMI by shRNA increases NF-κB transcriptional activity. Mechanistically, NMI associates with NF-κB/p65 and inhibits NF-κB/p65 nuclear translocation and thereby negatively regulates NF-κB/p65 transcriptional activity. Taken together, our results demonstrate that NMI modulates the NF-κB signaling pathway by sequestering NF-κB/p65 in the cytoplasm, resulting in reduced IL-6 and IL-1β production after TNF-α stimulation. Treatment with IFNα in the presence of NMI leads to increased apoptosis in tumor cells. These findings reveal a novel mechanism by which NMI regulates NF-κB activity.

## Introduction

Since the discovery of the NF-κB transcription factors in 1986 by Sen and Baltimore, many studies have shown the link between the NF-κB signaling pathway and the control of the cell responses, including infection, inflammation, apoptosis and the epithelial-mesenchymal transition^1–4^. The mammalian NF-κB family consists of five members: NF-κB 1(p50 and p105), NF-κB 2 (p52 and p100), RelA (p65), RelB and c-Rel. These proteins share a Rel homology domain that mediates DNA binding, dimerisation and interactions with specific inhibitory factors named IκBs, which retain NF-κB dimers in the cytoplasm^5,6^. The activation of the NF-κB signaling pathway by some stimuli, such as inflammatory agents, carcinogens, tumor promoters, viral proteins, stress, chemotherapeutic agents, and γ radiation results in the phosphorylation of the IκB subunit by activated IKKs, leading to the proteasome-mediated degradation of IκBs. IKK is composed of the IKKα, IKKβ, and IKKγ (NEMO) subunits. After activation, IKKβ phosphorylates IκBα on the serines at position 32 and 36, resulting in its polyubiquitination and proteasomal degradation, which are critical for NF-κB nuclear translocation. The activated NF-κB in the nucleus acts as a transcription factor that regulates the expression of numerous genes, including IL-6 and IL-1β^5,7–9^.

NF-κB is constitutively active in most tumor cell lines and various types of tumor tissues derived from patients^10–15^, which implicates the suppression of NF-κB as an important new approach for the treatment of a variety of cancers. Numerous proteins that can inhibit the NF-κB signaling pathway, such as A20, zinc finger protein inhibiting NF-κB (ZIN), p65-interacting inhibitor of NF-κB (SINK), ABIN1, ABIN2, KAP1, and protein inhibitor of activated STAT1 (PIAS1), were recently identified^3,9,16–24^. In this study, we identified NMI as an additional inhibitor of NF-κB activation triggered by TNF-α stimulation.

Originally identified as a N-Myc-interacting protein, NMI is an IFN-inducible protein that interacts with a variety of key transcription factors, such as c-Myc, N-Myc, Max, Tip60, c-Fos, Sox-10, IFP35, apoptin, breast cancer 1(BRCA1), CKIP-1, and STAT proteins with the exception of STAT2 protein^25–33^. In response to IL-2 and IFN**γ** stimulation, NMI enhances the association of the CBP/p300 coactivator proteins with STAT1 and STAT5, and in combination with CBP/p300 can augment STAT-mediated transcription^34^. NMI has also been reported to serve as a Sendai virus-inducible protein, and it can interact with Interferon regulatory 7 (IRF7), promote the K48-linked ubiquitination and the proteasome-dependent degradation of IRF7, which inhibits virus-triggered type I IFN production^30^. Another recent study showed that NMI interacts with viral Tas protein and suppresses Prototype Foamy Virus by sequestering Tas protein in the cytoplasm^35,36^. Taken together, these findings demonstrate that NMI plays an important role in immune regulation and inflammatory responses. NMI is also involved in the regulation of EMT and acts as a negative regulator of EMT. The loss of NMI promotes EMT through the activation of the TGF-β/SMAD signaling via the regulation of SMAD7^1^. Overexpression of NMI inhibits the Wnt/β-catenin signaling pathway through the upregulation of DKK1 and retards tumor cell growth^37^. In addition, NMI participates in the cell cycle control, DNA damage response and tumorigenesis^25–29,37–39^.

Previously, we showed that NMI promotes the interaction between NF-κB/p65 and histone deacetylases (HDACs) and inhibits the acetylation of p65. NMI suppresses tumor invasion and metastasis through the inhibition of NF-κB signaling^40^. In this study, we further investigated the mechanism that NMI regulates NF-κB activity. We found that NMI suppresses the transcriptional activity of NF-κB and reduces TNF-α-induced IL-6 and IL-1β production. Confocal microscopy analysis showed that p65 is sequestered by NMI in the cytoplasm after TNF-α stimulation, preventing the activation of NF-κB. Upon stimulation with apoptosis agents NMI-overexpressing cells exhibited a higher degree of cell death compared with control cells due to the inhibition of NF-κB cell survival signaling by NMI. Our results reveal a new mechanism by which NMI regulates NF-κB activity.

## Results

### NMI negatively regulates TNF-α-induced IL-6 production

**Figure 1:**
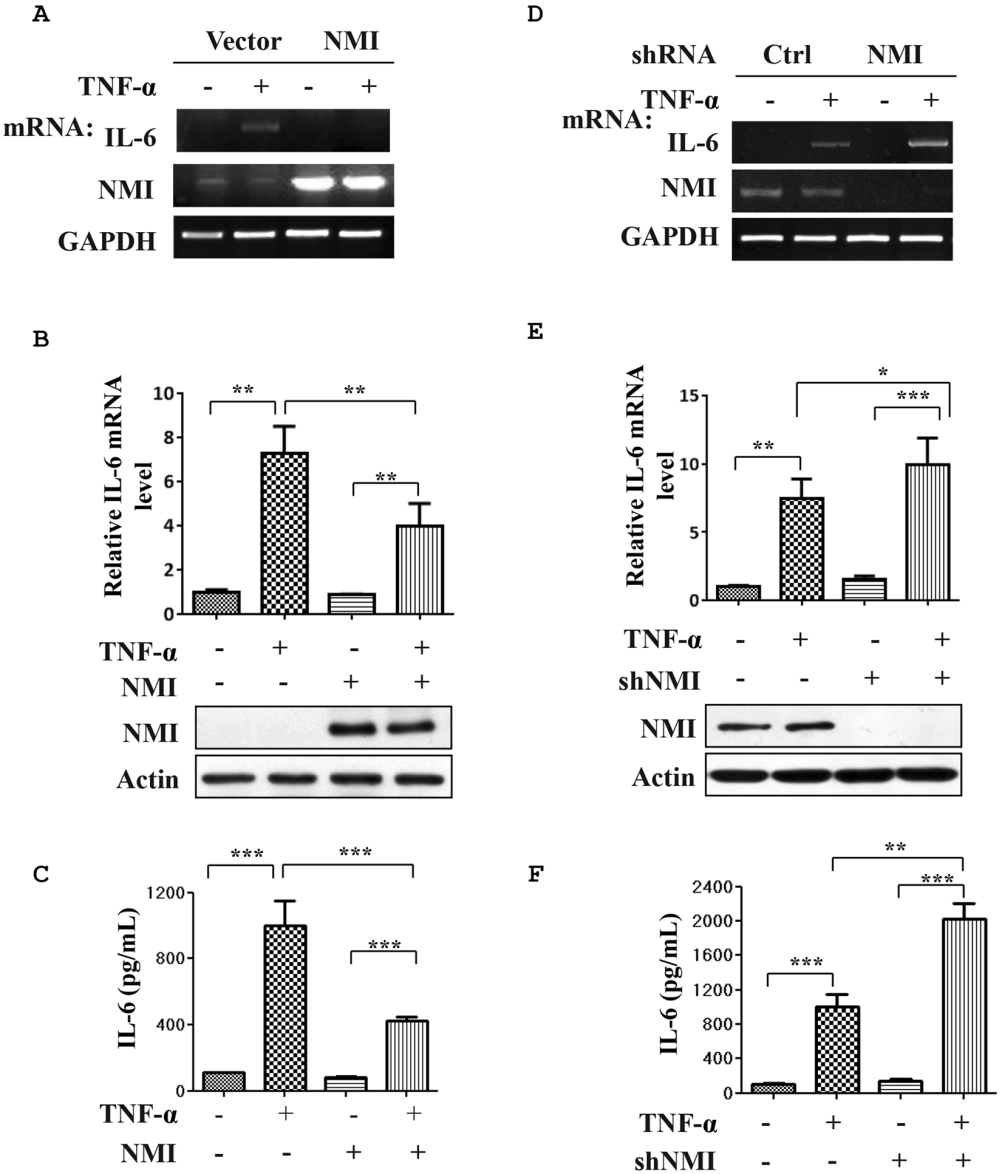
NMI negatively regulates TNF-α-induced IL-6 production. **(A)** HeLa cells in a 6-well plate were transfected with control or NMI-expression plasmids, and the cells were left untreated or treated with TNF-α (10 ng/ml) for an additional 2 h. The total RNA from these cells was isolated and subjected to qPCR analysis using IL-6, NMI, and GAPDH primers. **(B)** The IL-6 mRNA expression level was quantified by qPCR analysis. The data represent the level of IL-6 mRNA normalized to the level of GAPDH, which was used as an internal control, and are expressed relative to the level in the control-treated samples that were not stimulated with TNF-α. Results are representative of three independent experiments, and the error bars represent the SD. **p < 0.01. An aliquot of each total cell lysate (TCL) was analyzed by immunoblotting with anti-NMI or anti-actin Abs. **(C)** HeLa cells were transfected with control or NMI-expression plasmids, and the cells were left untreated or treated with TNF-α (10 ng/ml) for an additional 12 h. The IL-6 levels in the cell culture supernatants were assayed by ELISA. Results are representative of three independent experiments, and the error bars represent the SD. ***p < 0.001. **(D)** HeLa cells were infected with lentiviruses expressing either control or shRNA targeting NMI. After 48 h, the cells were left untreated or treated with TNF-α (10 ng/ml) for an additional 12 h. The total RNA samples isolated from these cells were subjected to qPCR analysis using IL-6, NMI, or GAPDH primers. **(E)** Analysis of IL-6 mRNA by quantitative RT-PCR. HeLa-shCtrl and HeLa-shNMI cell lines were left untreated or treated with TNF-α (10 ng/ml) for an additional 12 h, and the mRNA were extracted and subjected to quantitative RT-PCR. The quantified IL-6 transcript levels are shown. The whole-cell extracts were subjected to western blotting using anti-NMI or anti-actin Abs. **(F)** HeLa-shCtrl and HeLa-shNMI cell lines were left untreated or treated with TNF-α (10 ng/ml) for an additional 12 h. The IL-6 levels in the cell culture supernatants were assayed by ELISA. Results are representative of three independent experiments, and the error bars represent the SD. *p < 0.05, **p < 0.01, ***p < 0.001.

Recent studies have demonstrated that NMI regulates STAT-mediated transcription in response to some cytokines stimuli, suggesting a broader role for NMI in cytokine signaling^34^. This finding leads us to investigate the effects of NMI on proinflammatory cytokines, such as TNF-α signaling. To these end, HeLa cells in a 6-well plate were transfected with control or NMI expressing plasmids, and the cells were left untreated or treated with TNF-α (10 ng/ml) for 2 h. The cell lysates and total RNA samples were analyzed by western blotting and qPCR, respectively (Fig. 1A). Interestingly, NMI-overexpressing cells showed a reduced TNF-α-induced IL-6 mRNA level compared with the control cells (Fig. 1A,B). At the protein level, The TNF-α-induced production of the IL-6 protein was also decreased in the NMI-transfected HeLa cells (Fig. 1C). To further determine whether NMI is involved in the regulation of IL-6 induction, we silenced endogenous NMI expression in HeLa cells using shRNA. After infection with lentiviruses expressing either control (shCtrl) or shRNA targeting NMI (shNMI) for 72 h, HeLa cell lysates and total RNA samples were analyzed by western blotting and qPCR, respectively. As demonstrated in Fig. 1D–F, NMI-knockdown HeLa cell lines (HeLa-shNMI cells) showed enhanced TNF-α-induced mRNA levels and production of IL-6 compared with the control shRNA transfected cells (HeLa-shCtrl). Therefore, these results suggest that NMI suppresses TNF-α-induced cytokine production.

### NMI inhibits TNF-α induced NF-κB activation

**Figure 2:**
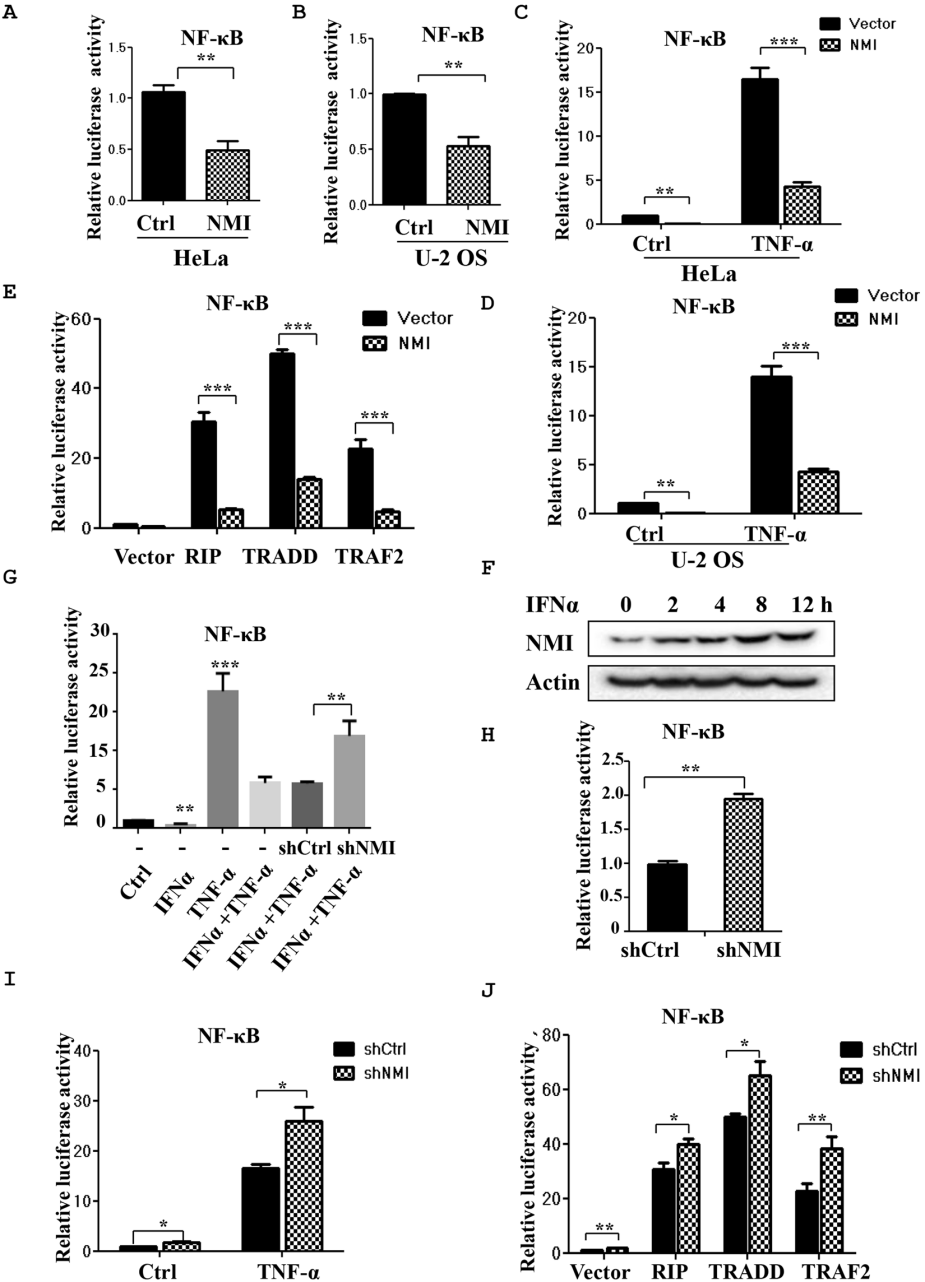
NMI inhibits TNF-α-induced NF-κB activation and knockdown of NMI via shRNA promotes TNF-α-induced NF-κB activation. **(A)** and **(B)** NF-κB luciferase reporter assays. HeLa cells (A) or U-2 OS cells (B) in 6-well plates were transiently transfected with a NF-κB luciferase reporter or a NF-κB luciferase reporter together with the FLAG-NMI plasmid. At 24 h posttransfection, the cells were harvested, and the cell lysates were subjected to luciferase assays. The average of the results from three independent experiments is shown. **p < 0.01. **(C)** and **(D)** NF-κB luciferase reporter assays. HeLa cells (C) or U-2 OS cells (D) in 6-well plates were transiently transfected with a NF-κB luciferase reporter or a NF-κB luciferase reporter together with the FLAG-NMI plasmid. At 24 h posttransfection, the cells were either untreated or treated with TNF-α for 6 h, and the cell lysates were subjected to luciferase assays. **(E)** NMI inhibits NF-κB activation induced by RIP, TRADD, or TRAF2. HeLa cells were transfected with the NF-κB luciferase reporter, the RIP/TRADD/TRAF2 plasmids and either control or FLAG-NMI. At 24 h posttransfection, the cell lysates were subjected to luciferase assays.** (F)** IFNα inhibits NF-κB activity. HeLa cells were transfected with the NF-κB luciferase reporter, and at 24 h posttransfection, the cells were treated as indicated for 6 h (TNF-α: 10 ng/ml, IFNα: 1000 U/ml). The cell lysates were subjected to luciferase assays and western blot analysis.** (G)** IFNα induces the expression of NMI. HeLa cells were treated with IFNα (1000 U/ml) for the indicated times, and the cell lysates were subjected to western blot analysis with anti-NMI Ab. **(H)** HeLa-shCtrl or HeLa-shNMI cells were transiently transfected with a NF-κB luciferase reporter. At 24 h posttransfection, the cells were harvested, and the cell lysates were subjected to luciferase assays. **(I)** HeLa-shCtrl or HeLa-shNMI cells were transfected with a NF-κB luciferase reporter. At 24 h posttransfection, the cells were either untreated or treated with TNF-α for 6 h, and the cell lysates were subjected to luciferase assays. **(J)** HeLa-shCtrl or HeLa-shNMI cells were transfected with a NF-κB luciferase reporter or the NF-κB luciferase reporter together with the RIP/TRADD/TRAF2 plasmids as indicated, and the cell lysates were then subjected to luciferase assays. All the data shown are the averages of the results from three independent experiments. The data are presented as the mean ± SD of duplicates. *p < 0.05, **p < 0.01, ***p < 0.001.

The induction of IL-6 after TNF-α stimulation is largely dependent on NF-κB. Therefore, we examined the effects of NMI on the transcriptional activation of NF-κB using a transient reporter assay. HeLa and U-2 OS cells were transiently transfected with the NF-κB-LUC construct together with the FLAG-NMI plasmid. At 24 h posttransfection, the cells were collected and subjected to a reporter assay. As shown in Fig. 2A, the NF-κB-mediated gene activation was inhibited by NMI, which caused a two-fold decrease in NF-κB-mediated gene expression. Similar results were observed in human osteosarcoma U-2 OS cells (Fig. 2B). Next, we treated HeLa cells (Fig. 2C) or U-2 OS cells (Fig. 2D) with TNF-α and examined the NF-κB-LUC activity in the presence or absence of NMI. As shown in Fig. 2C,D, TNF-α stimulation induced strong NF-κB-LUC activity, and expression of NMI significantly inhibited the acivity of NF-κB in Hela or U-2 OS cells after TNF-α stimulation. These results suggest that TNF-α-induced activation of NF-κB. To further verify this conclusion, we examined the mRNA levels and synthesis of IL-1β, another NF-κB-dependent inflammatory gene in the NMI overexpressing cells or NMI-silenced cells after TNF-α stimulation. The results (Supplementary Fig. S1) revealed that NMI inhibits the TNF-α induced production of IL-1β.

Overexpression of the p55 TNF-α receptor or its downstream signaling proteins receptor-interacting protein (RIP), TNF receptor-associated death domain (TRADD) and TNF receptor-associated factor 2 (TRAF2) can activate NF-κB transcription^17,20,41^. We found that NMI also inhibits NF-κB activation induced by RIP, TRAF2, or TRADD overexpression in HeLa cells (Fig. 2E). It is known that IFNα treatment can induce the expression of NMI^29^ and we validated this IFNα-mediated NMI induction in HeLa cells (Fig. 2F). We therefore assessed IFNα treatment on TNF-α-induced NF-κB activation. HeLa cells were transiently transfected with NF-κB-LUC, and 24 h posttransfection the cells were left untreated or treated with IFNα (1000 U/ml) and TNF-α (10 ng/ml) as indicated and cell lysates were subjected to luciferase assays. As shown in Fig. 2G, IFNα treatment significantly inhibited the TNFα-induced NF-κB activation. This IFNα-mediated decrease was partially abrogated in NMI knockdown cells, suggesting that IFNα suppresses NF-κB activation possibly through induced NMI expression.

Next, we investigated whether knockdown of NMI with RNAi upregulates TNF-α stimulated NF-κB activation. As shown in Fig. 2H and I, suppression of NMI with shRNA enhanced TNF-α stimulated NF-κB-LUC activity in HeLa-shNMI cells.

In accordance with these results, shNMI also increased NF-κB activation induced by RIP, TRADD and TRAF2 overexpression in HeLa cells (Fig. 2E). These results demonstrated that NMI inhibits TNF-α-stimulation NF-κB activation.

### NMI does not affect IκBα degradation

**Figure 3:**
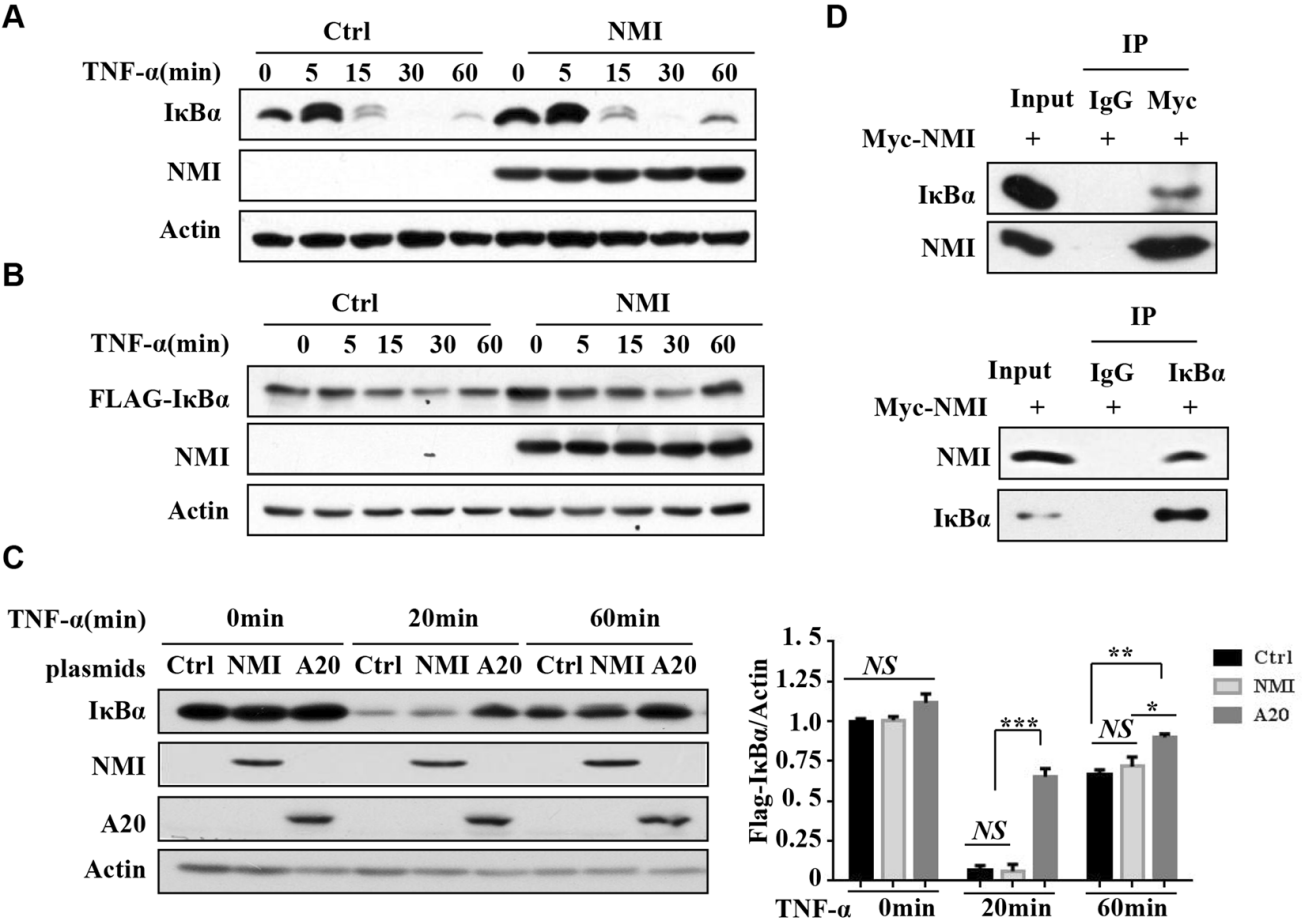
NMI does not affect the degradation of IκBα. **(A)** HeLa cells were transfected with the control or Myc-NMI plasmids. At 24 h posttransfection, the cells were treated with TNF-α (10 ng/ml) for the indicated periods. The cell extracts were subjected to western blotting using the indicated Abs. **(B)** HeLa cells were transfected with the FLAG-IκBα and Myc-NMI plasmids as indicated. At 24 h post transfection, the cells were treated with TNF-α (10 ng/ml) for the indicated times. The cell extracts were subjected to western blotting using the indicated Abs. **(C)** HeLa cells were transfected with the control, Myc-NMI or A20 plasmids. At 24 h posttransfection, the cells were treated with TNF-α (10 ng/ml) for the indicated periods. The cell lysates were immunoblotted with the indicated Abs. Statistical analysis results of IκBα expression was in the right panel. Actin was used as the internal control. The results are representative of three independent experiments, and the error bars represent the SD. *p < 0.05, **p < 0.01, ***p < 0.001. **(D)** NMI binds to IκBα. 293 T cells were transfected with the Myc-NMI plasmid, and the cell lysates were immunoprecipitated with anti-Myc or anti-IκBα Ab and immunoblotted for the indicated proteins.

The degradation of IκBα is required for NF-κB activation. To delineate the molecular mechanisms of NMI-mediated downregulation of TNF-α-induced NF-κB activation, we examined the effect of NMI on\ TNF-α-induced IκBα degradation. As shown in Fig. 3A, overexpression of NMI had no significant effect on TNF-α-induced IκBα degradation. We also constructed FLAG-tagged IκBα expression plasmid and examined TNF-α-induced degradation of ectopically expressed Flag-IκBα protein. Overexpression of NMI also did not affect the result TNF-α-stimulated FLAG-IκBα degradation (Fig. 3B). In contrast, A20, a zinc finger protein that exerts inhibitory effects on TNF signaling^41^, significantly inhibited TNF-α-stimulated IκBα degradation (Fig. 3C and Supplementary Fig. 4). Therefore, NMI does not affect events upstream of IκBα degradation. Interestingly, we found that overexpressed NMI associated with IκBα in 293 T cells (Fig. 3D), although GST-pull down assay suggesting that this interaction is indirect (data not shown). In conclusion, although NMI interacts with IκBα, it does not affect IκBα degradation.

### NMI interacts with members of NF-κB family protein

**Figure 4:**
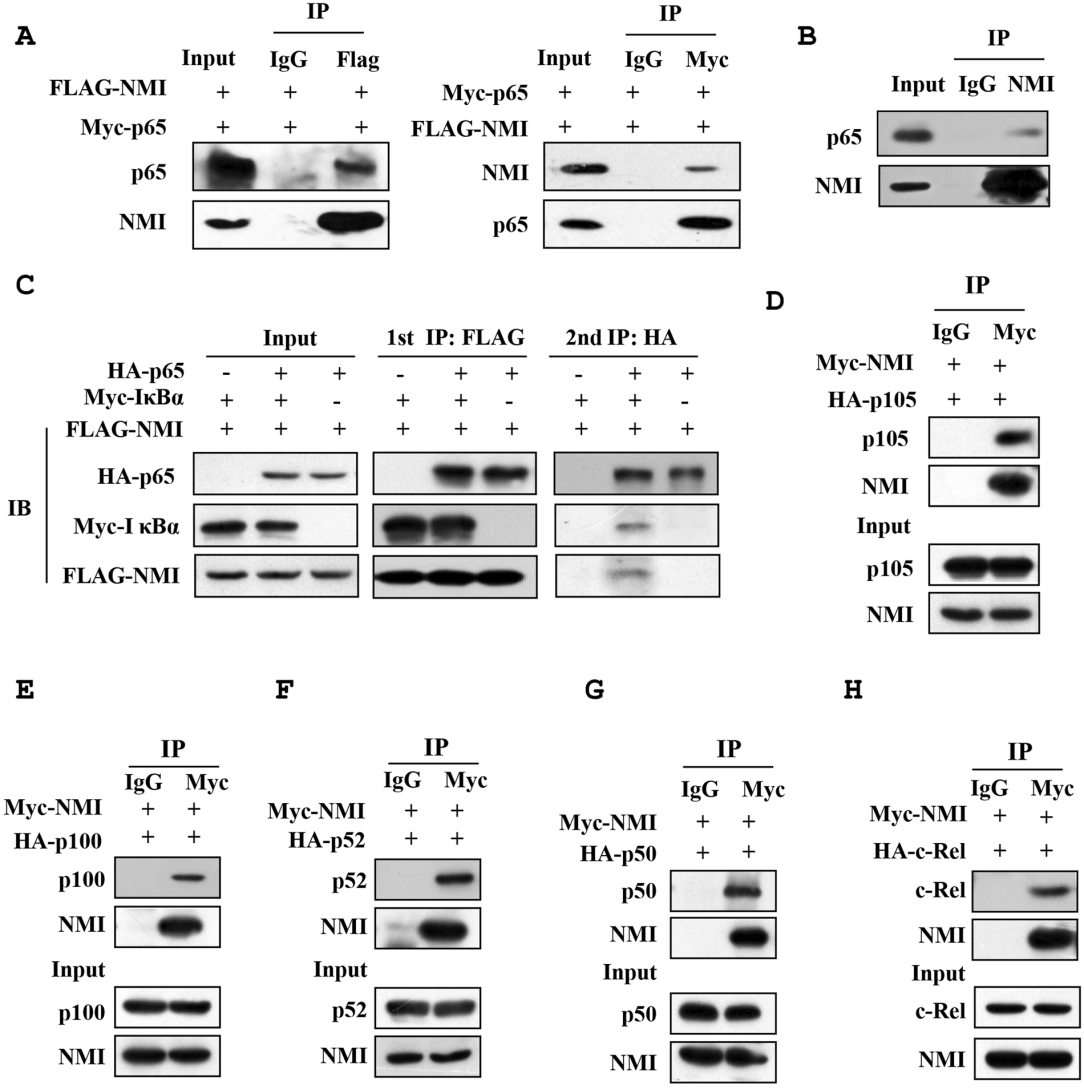
NMI interacts with members of NF-κB family protein. **(A)** The 293 T cells were transfected with the Myc-p65 and FLAG-NMI plasmids. At 24 h posttransfection, the cells were lysed, immunoprecipitated with anti-FLAG (left panel) or anti-Myc (right panel) Abs, and immunoblotted with anti-Myc or anti-FLAG Abs. An aliquot of each total cell lysate was analyzed by immunoblotting with the indicated Abs. **(B)** Endogenous NMI interacts with p65. HeLa cells were lysed, the cell lysates were then immunoprecipitated with anti-NMI Ab, and immunoblotted with anti-p65 Ab. **(C)** Two-step co-IP assay. The HEK293T cells were transfected with the HA-p65, FLAG-NMI and Myc- IκBα plasmids as indicated. Cells were lysed. The first immunoprecipitation was performed with an anti-FLAG antibody. The complex was eluted with the FLAG peptide, followed by the second step of immunoprecipitation with an anti-HA antibody. Protein samples from each step were subjected to western blot analysis. **(D–H)** NMI interacts with members of NF-κB family proteins. HeLa cells were transfected with the indicated plasmids, and the cells were lysed, immunoprecipitated with anti-Myc Ab, and immunoblotted with anti-HA Ab.

Previously, it has been demonstrated that NMI interacts with various transcription factors and regulates their function^25–33^, therefore, we investigated whether NMI binds to the NF-κB family proteins by Co-IP assay. As shown in Fig. 4A, Myc-p65 can be clearly observed in the FLAG-NMI but not the control immunoprecipitates. In a reciprocal Co-IP experiment, FLAG-NMI was readily detected in the Myc-p65 immunoprecipitates. We also found that endogenous NMI associates with p65 (Fig. 4B). These data demonstrated that NMI interacts with p65. Next, we examined whether NMI binds to other NF-κB family proteins, including p100/52, p105/p50 and c-Rel. The Co-IP assays suggested that NMI interacts with all these proteins (Fig. 4D–F). We suspected that NMI may form a ternary complex with NF-κB and IκBα. To test this hypothesis, we performed a two-step Co-IP. As shown in the Fig. 4C, the results of two-step Co-IP assay showed that these three proteins are indeed in a ternary complex.

### NMI inhibits the nuclear translocation of NF-κB/p65

**Figure 5:**
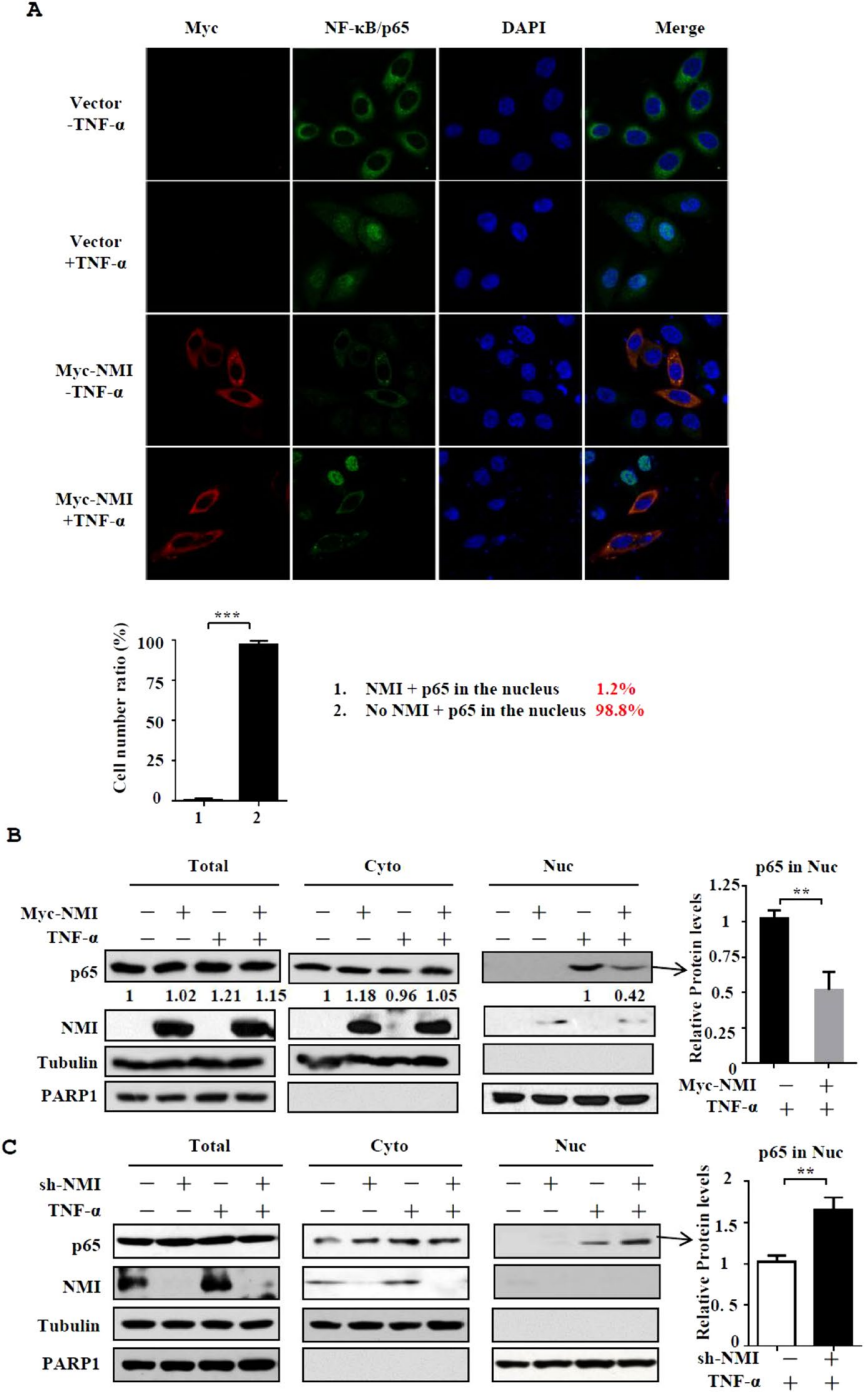
NMI inhibits the nuclear translocation of NF-κB/p65 after TNF-α stimulation. **(A)** HeLa cells were transfected with control or Myc-NMI plasmids, and at 24 h posttransfection, the cells were left untreated or treated with TNF-α (10 ng/ml) for 60 min. The cells were fixed and incubated with anti-p65 or anti-Myc Ab and then stained with rhodamine-conjugated anti-mouse IgG (red) or FITC-conjugated anti-rabbit Ab (green). The same slide was also stained with DAPI to show the nucleus. The expression and localization of p65 and Myc-NMI was determined by confocal immunofluorescence analysis. The percentage of the cells expressing p65 in the nucleus among the cells that express Myc-NMI and the cells that don’t express Myc-NMI was calculated in the bottom panel. **(B)** HeLa cells were transfected with control or Myc-NMI plasmids, and at 24 h posttransfection, the cells were left untreated or treated with TNF-α (10 ng/ml) for 60 min. The cells were then harvested and fractionated into the nuclear and cytoplasmic fractions, and the fractions were immunoblotted with anti-NMI and anti-p65 Abs. The quantification by densitometry of western blots of p65 bands without or with Myc-NMI in TNF-α-stimulated nuclear fractions was shown in the right panel. The results are representative of three independent experiments, and the error bars represent the SD.**p < 0.01. **(C)** HeLa shCtrl and HeLa shNMI cells were treated in (B), and performed the cellular fractionation assay.

Our results demonstrated that NMI associates with NF-κB and IκBα, but NMI does not affect the degradation of IκBα. This findings suggest that NMI regulates NF-κB activation though other mechanisms. We examined whether NMI affects the nuclear translocation of p65 after TNF-α stimulation using confocal microscopy. As shown in the Fig. 5A, after TNF-α stimulation the majority of p65 translocate into the nucleus. Interestingly, in NMI overexpressing cells, p65 colocalizes with NMI in the cytoplasm, and p65 nuclear translocation did not occur after TNF-α stimulation. To confirm this result, we performed cellular fractionation assay. Western blotting analysis showed that significantly less p65 was translocated to the nuclear fraction after TNF-α stimulation in NMI-overexpressing HeLa cells compared with the control cells (Fig. 5B). Conversely, downregulation of NMI expression in HeLa cells with shRNA increases the nuclear translocation of p65 (Fig. 5C). These data suggested that NMI inhibits NF-κB activation by affecting the nuclear translocation of NF-κB/p65 in response to TNF-α stimulation. To see whether NMI-mediated cytoplasmic sequestration of NF-κB/p65 is dependent on IκBα, we knockdowned IκBα expression with siRNA and analyzed the p65 nuclear translocation in NMI-expressing HeLa cells after TNF-α stimulation. The results showed that IκBα knockdown has no effect on NMI-mediated sequestration of NF-κB/p65 in the cytoplasm (Supplementary Fig. 3).

### NMI enhances TNF-α triggered apoptosis

**Figure 6:**
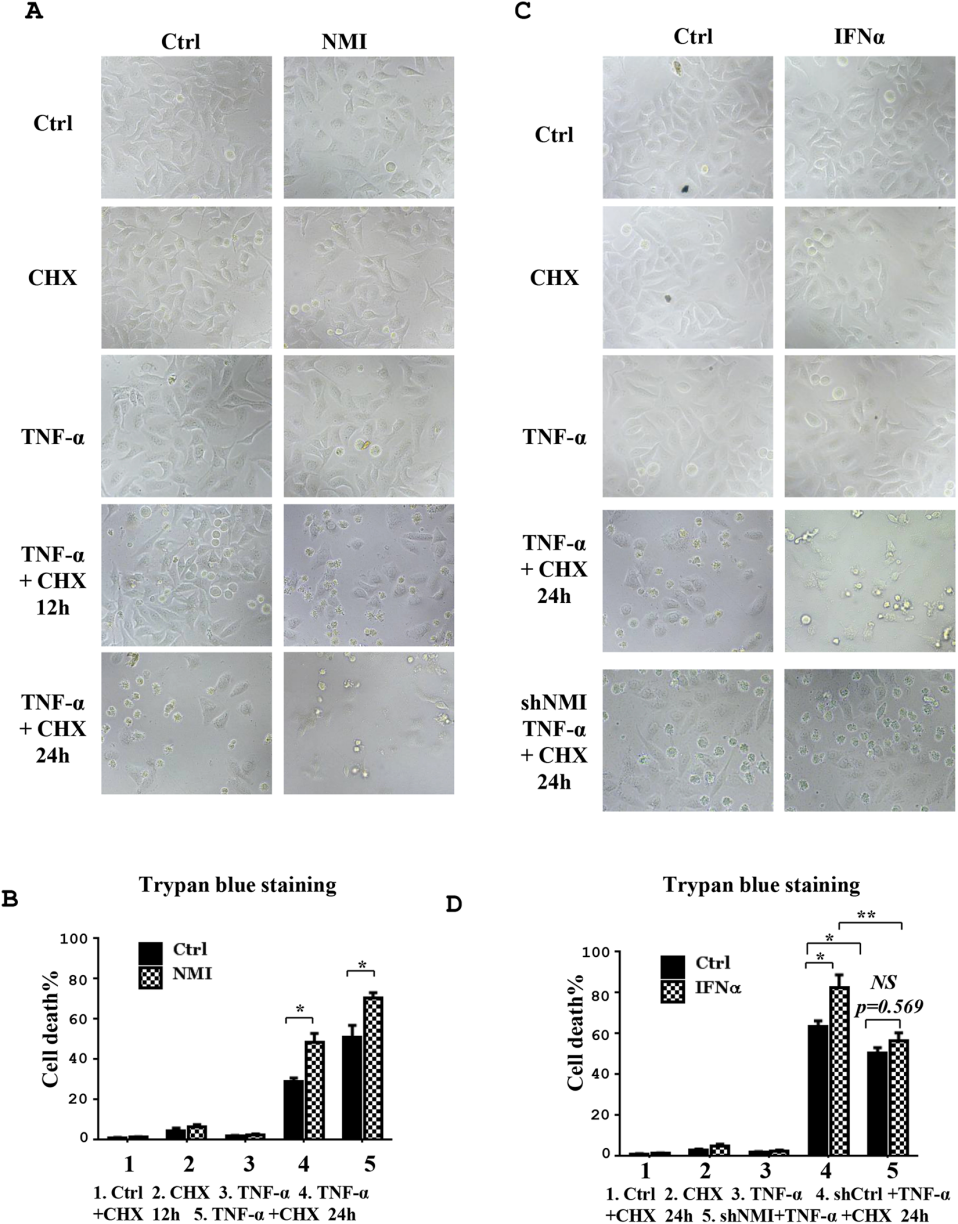
NMI enhances TNF-α plus CHX induced HeLa cell apoptosis. **(A)** The NMI-overexpressing HeLa cells (HeLa-NMI) showed an increased apoptotic morphology after TNF-α plus CHX treatment. HeLa-Ctrl and HeLa-NMI cells were plated in 12-well plates. The following day, the cells were treated as indicated (TNF-α: 5 ng/ml, CHX: 5 μg/ml) for 12 h or 24 h, and the cells images were then taken with a Nikon-TE2000 microscope. (**B**) The cell death in (A) was quantified by a Trypan blue staining assay. The data are the mean ± S.D. of three independent experiments. *p < 0.05. **(C)** IFNα increases the apoptosis induced by TNF-α plus CHX in HeLa cells. HeLa cells were plated in 12-well plates, and the next day the cells were untreated or treated with IFNα for 12 h and then treated with TNF-α plus CHX for the indicated times (TNF-α: 5 ng/ml, CHX: 5 μg/ml). **(D)** The ratio of cell death in (C) was determined by a Trypan blue staining assay. The results are representative of three independent experiments, and the error bars represent the SD. *p < 0.05, **p < 0.01.

**Figure 7:**
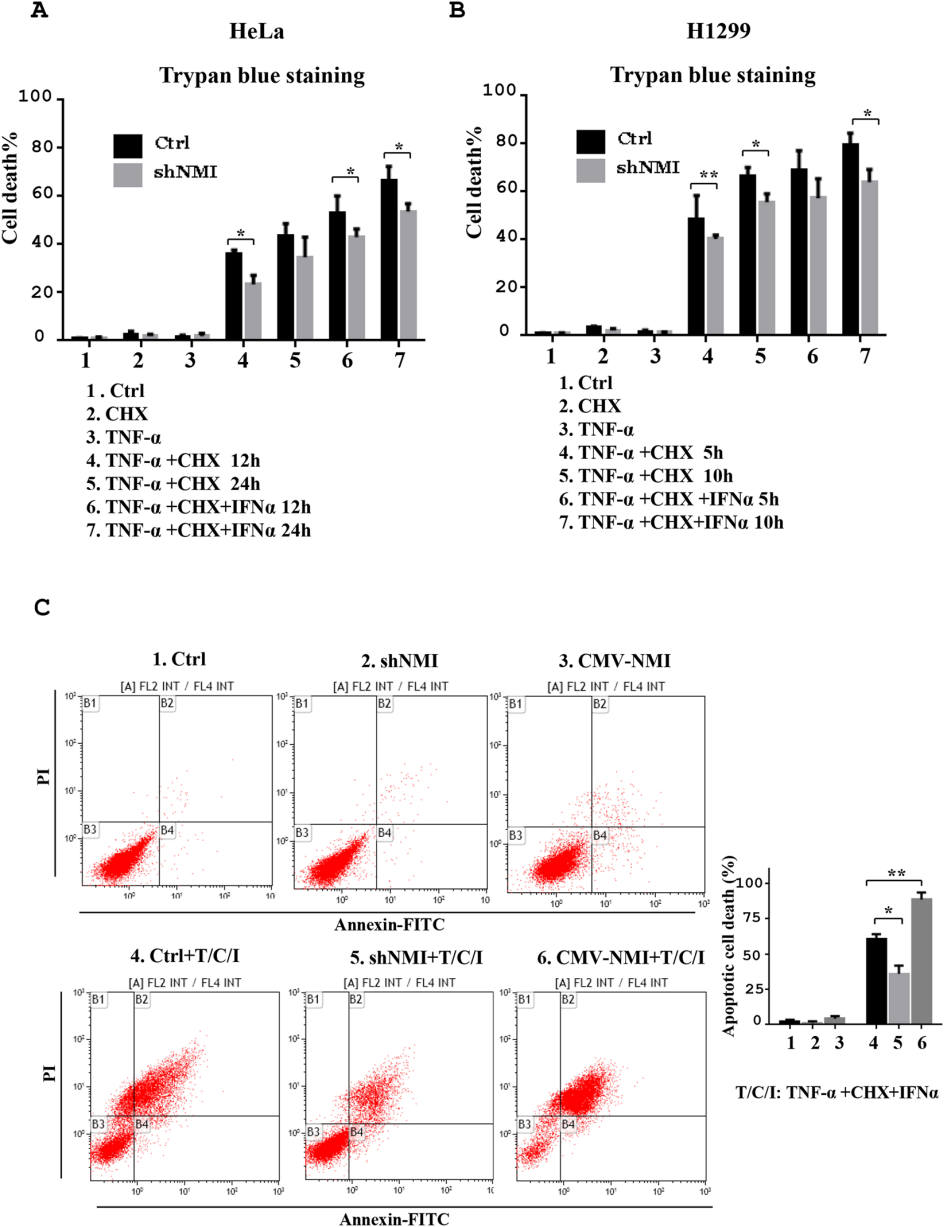
Knockdown of NMI inhibits TNF-α plus CHX stimulated cell death. **(A)** HeLa-shCtrl and HeLa-shNMI cells were plated in 12-well plates. The following day, the cells were treated as indicated (TNF-α: 5 ng/ml, CHX: 5 μg/ml, IFNα:1000U/ml), and the cells were obtained and the ratio of cell death was determined with a Trypan blue staining assay. The results are representative of three independent experiments, and the error bars represent the SD.*p < 0.05. **(B)** H1299-shCtrl and H1299-shNMI cells were treated as in (A), and cell death was analysed by a Trypan blue staining assay. The results are representative of three independent experiments, and the error bars represent the SD.*p < 0.05, **p < 0.01. **(C)** FACS analysis. HeLa-shCtrl, HeLa-shNMI and HeLa-NMI cells were untreated or treated with IFNα for 12 h and then treated with TNF-α plus CHX for the indicated times (TNF-α: 5 ng/ml, CHX: 5 μg/ml). The cells were harvested, stained with propidium iodide (PI) and annexin V -FITC analyzed by flow cytometry. Data are represented as means ± s.e.m. P-values were calculated using Student’s t-test. *p < 0.05, **p < 0.01.

Since NF-κB mediated expression of anti-apoptosis genes is requried for cell survival^29^, we speculate that the inhibition of NF-κB activation by NMI will increase TNF-α induced cell death. To test this hypothesis, we treated control and NMI-overexpressing HeLa cells with apoptotic trigger TNF-α plus CHX for 12 or 24 h. Compared to control cells, NMI-overexpressing HeLa cell lines presented an increased apoptotic cell death (Fig. 6A and B). As it has been demonstrated that IFNα induced the expression of NMI^29^, we test whether IFNα treatment increases the TNF-α stimulation death. Indeed, TNF-α plus CHX induced more cell death in the presence of IFNα (Fig. 6C and D). Knockdown of NMI with shRNA impaired the effect of IFNα on TNF-α-stimulated apoptosis (Figs 6C,D and 7A,C), confirming that IFNα-induced expression of NMI enhances TNF-α triggered cell killing.

Similar results were obtained with another cell line H1299 (Fig. 7A,B and Supplementary Fig. S2A–D). Taken together, these results indicate that the induction of NMI may be the mechanism through which IFN-α inhibits the activation NF-κB and cell survival.

## Discussion

NF-κB is a key regulator of gene transcription involved in immune, inflammation and stress responses. Recent studies have revealed that the regulation of NF-κB is more complex than previously believed, and an increasing number of proteins that affect the NF-κB transcriptional activities and consequently influence cellular progress continue to be identified.

As an IFN-inducible protein NMI interacts with a variety of key transcription factors, such as c-Myc, N-Myc, Tip60, Max, IFP35, Sox-10, c-Fos, apoptin, breast cancer 1(BRCA1), CKIP-1, and STAT proteins with the exception of STAT2 protein^25–33^. By interacting with these proteins, NMI participates in the cell cycle control, apoposis, DNA damage response and tumorigenesis^25–29,37–39^. NMI is also involved in the regulation of EMT and acts as a negative regulator of EMT. The loss of NMI promotes EMT through the activation of the TGF-β/SMAD signaling and Wnt/β-catenin signaling pathway^1,37^. NMI has also been reported to serve as a Sendai virus-inducible protein and interacts with Interferon regulatory 7 (IRF7) to inhibits virus-triggered type I IFN production^30^. Another recent study showed that NMI interacts with viral Tas protein and suppresses Prototype Foamy Virus^35^. Taken together, these findings demonstrate that NMI plays an important role in immune regulation and inflammatory responses.

**Figure 8:**
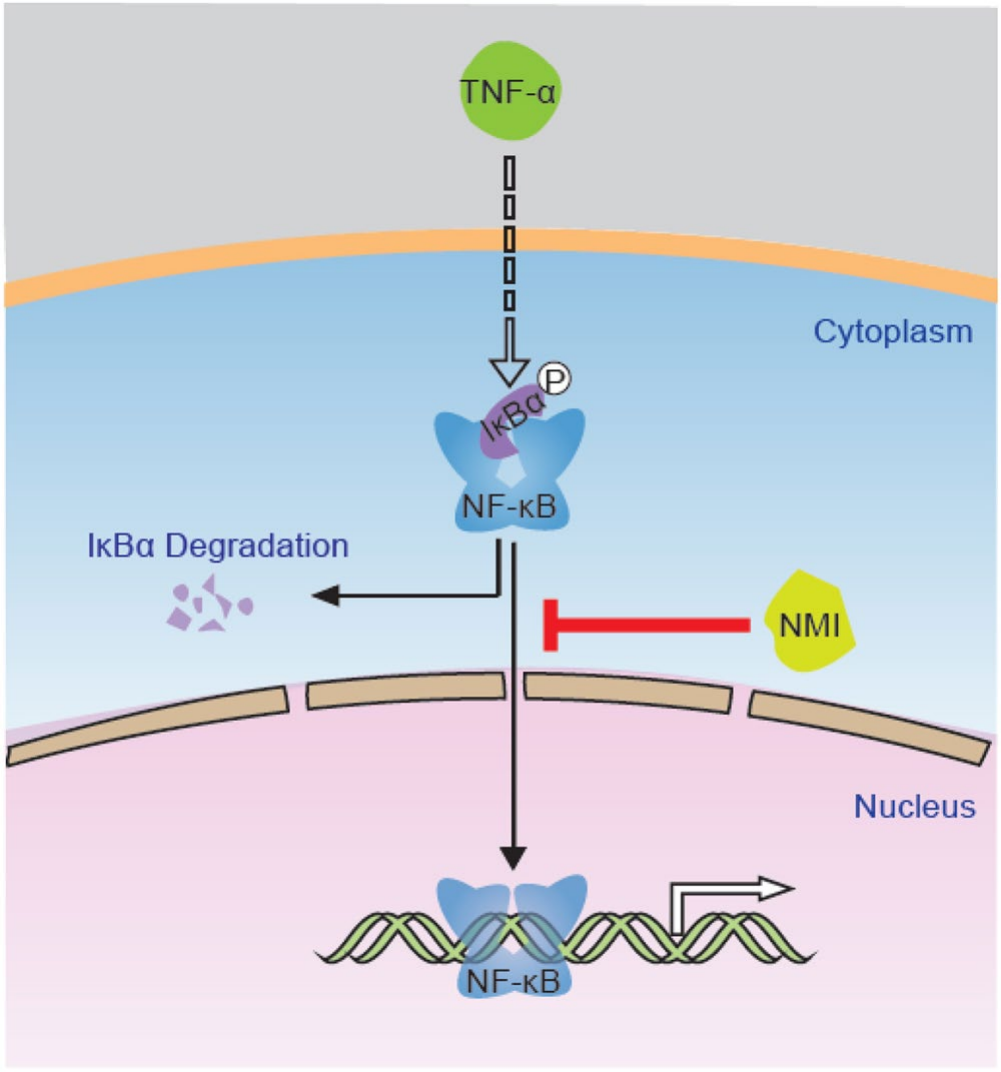
Working model of NMI in the regulation of NF-κB activity. NMI inhibits NF-κB activity by affecting the nuclear translocation of NF-κB/p65.

In this study, we identified NMI as a novel regulatory component of TNF-α/NF-κB signaling pathway. We demonstrated that NMI suppresses TNF-α-induced IL-6 and IL-1β production. Ectopic expression of NMI inhibits NF-κB-mediated transcriptional activities. We showed that NMI associates with NF-κB/p65 and inhibits NF-κB/p65 nuclear translocation, thus negatively regulates NF-κB transcriptional activity. Through inhibition of NF-κB cell survival signaling, NMI enhances TNF-α stimulated apoptosis. Treatment with IFNα induced the expression of NMI, which consequently results in increased TNF-α stimulated cell death. Our results indicate that NMI is a negative regulator (Fig. 8).

Under most circumstance, inducible NF-κB activation is rapidly attenuated^2,24,42–44^. Several proteins that inhibit NF-κB activation pathways, such as A20, zinc finger protein inhibiting NF-κB (ZIN), p65-interacting inhibitor of NF-κB (SINK), ABIN1, ABIN2, KAP1, and protein inhibitor of activated STAT1 (PIAS1), have been identified^3,9,16–24^. In this study, we identified NMI as an additional inhibitor of NF-κB signaling.

In normal cells, NF-κB remains in the cytoplasm bound to inhibitory proteins known as IκBs (inhibitors of NF-κB). Upon stimulation, IκB proteins are phosphorylated, ubiquitinated and degraded. The released NF-κB then translocates to the nucleus and upregulates gene expression^45,46^. Besides IκB proteins degradation, posttranslational modification such as phosphorylation and acetylization regulate NF-κB/p65 activities. Although our results suggest that NMI inhibits NF-κB/p65 nuclear translocation, the mechanism by which NMI affects the activity of p65 exist. Our latest study showed that NMI promotes the interaction between NF-κB/p65 and histone deacetylases (HDACs) and inhibits the acetylation and transcriptional activity of p65, thus inhibiting cell migration and invasion in stomach neoplasm. A recent study indicated that STAT3 enhances the acetylation of NF-κB/p65^47^. The STAT3-mediated enhancement of NF-κB/p65 acetylation requires serine and tyrosine phosphorylation^9,48,49^. We found that NMI can interact with STAT3 (data not shown), which is consistent with previous studies^34^. Since STAT3 also interacts with p65, NMI might compete with STAT3 for binding to p65. Future studies are needed to test this hypothesis.

In conclusion, we found that NMI negatively regulates TNF-α-stimulated IL-6 and IL-1β production in HeLa cells and inhibits TNF-α-stimulated NF-κB transcriptional activity by sequestering NF-κB in the cytoplasm. We identified NMI as an important negative regulator of NF-κB, providing new insights into the molecular mechanisms that control NF-κB activation and inflammation.

## Materials and Methods

### Plasmids

Full-length cDNA encoding human NMI was obtained by PCR and subcloned into pCMV-*HA/Myc*, pCMV-*FLAG* (Clontech) and pLL3.7-CMV vectors. GST-NMI plasmid was constructed by PCR and subcloning into pGEX-4T vector (Clontech). All constructs derived from PCR products were verified by DNA sequencing. pCMV-*HA/Myc-p65* were kindly provided by professor Jiahuai Han (Xiamen University, Xiamen, China). Luciferase reporter plasmid pGL-3-*NF-κB*-*LUC* and GST-IκBα were from Dr.Chundong Yu (Xiamen University, Xiamen, China).

### RNA interference

For RNA interference experiments we used a lentivirus-based vector, pLL3.7 (Clontech). Oligonucleotides targeting NMI (GenBank accession number: U32849) (NMI shRNA-1, 5′-GAGGACAGTGCTTCTGACA-3′; NMI shRNA-2, 5′-GGAGCATTCGCCAGATGAA-3′) were cloned into the pLL3.7 vector. Recombinant lentiviral plasmids were co-transfected into 293 T cells with the packaging plasmids VSV-G, RSV-REV and pMDL. After 48 h the viral supernatants were passed through 0.45-μm filters and used to infect target cells in the presence of 6 μg/ml polybrene (Sigma-Aldrich).

### Cell culture, DNA transfection and treatments

HEK293T, U-2 OS, HeLa and H1299 cells were cultured in DMEM (Life Technologies, Carlsbad, CA, USA) supplemented with 10% fetal bovine serum (Hyclone, Logan, UT, USA) and 100 μg/ml penicillin and streptomycin. Transient transfections were carried out using the standard calcium phosphate method. TNF-α was purchased from PeproTech (PeproTech EC), and IFNα was obtained from Chinese Academy of Sciences. The reagents were added to subconfluent cells at the indicated doses.

### Immunoprecipitation (IP) and Western Blotting

Total cell extracts were prepared in cell lysis buffer (20 mM Tris-HCl [pH7.5], 150 mM NaCl, 20 mM β-glycerophosphate, 10 mM NaF, 1 mM sodium orthovanadate, 1 mM PMSF, 2 μg/ml aprotinin, 10 μg/ml leupeptin, 1% Triton X-100, and 1 mM EDTA) for immunoprecipitations. Immunocomplexes were resolved by SDS-PAGE, and Western blotting was performed with the following Abs: anti-FLAG M2 and anti-actin monoclonal Abs (Sigma-Aldrich); anti-HA (Y-11), anti-IκB (C-20) and anti-NMI (N-16) polyclonal Abs, anti-Myc (9E10), anti-GFP (B-2) mAb (Santa Cruz Biotechnology); NF-κB/p65 (L8F6) mAb (Cell signaling); horseradish peroxidade-conjugated goat anti-mouse, goat anti-rabbit and donkey-anti goat (Thermo Scientific, Waltham, MA), and FITC-conjugated anti-rabbit IgG or rhodamine-conjugated anti-mouse IgG (Millipore).

### GST pull-down assay

GST-NMI and GST were expressed in *E*.*coli* and purified on Glutathione-Sepharose (GE Healthcare, Piscataway, NJ). Lysates from HeLa cells were prepared and incubated with GST or GST-NMI immobilized on Glutathione-Sepharose beads. The beads were washed five times with binding buffer, 1 × SDS loading buffer was added and analyzed by western blotting using the indicated Ab.

### RNA isolation, quantitative real-time PCR, and IL-6 and IL-1β quantification

Total RNA was isolated from cells using RNA Simple Total RNA Kit (Bioteke, Beijing, China), and first-strand cDNA was synthesized from 1 μg of total RNA using the First-strand cDNA Synthesis kit (Bioteke, Beijing, China) following the manufacturer’s instructions. Prepared cDNA samples were amplified and analyzed by quantitative real-time RT-PCR with Power SYBR Green PCR Master Mix (Applied Biosystems, Foster City, CA) using ABI 7500 Real Time PCR System (Applied Biosystems). Primer sequences were as follows: human IL-6, 5′-ACAACCACGGCCTTCCCTAC-3′(forward) and 5′-TCTCATTTCCACGATTTCCCAG-3′(reverse); human IL-1β, 5′GAGCTGAAAGCTCTCCACCTCA-3′(forward) and 5′-CATGAAGGAGAACCAAGCAACGA-3′(reverse); human GAPDH, 5′-GACATCAAGAAGGTGGTGAA-3′(forward) and 5′-TGTCATACCAGGAAATGAGC-3′(reverse). To quantify IL-6 and IL-1β production, the cells were stimulated with TNF-α for 12 h, and the IL-6 and IL-1β levels in the culture supernatants were then measured by ELISA Kit (BD Biosciences; Catalog #KHC0062) according to the manufacturer’s instructions.

### Indirect immunofluorescence microscopy

HeLa cells were seeded onto sterile coverslips in a 6-well plate at 30–40% confluence. The next day, plasmid transfection was performed as indicated. At 24 h post-transfection, the cells were washed with PBS, fixed in 4% paraformaldehyde for 20 min and permeabilized with 0.2% Triton X-100 for 10 min. After blocking for 30 min with blocking buffer (1% BSA in PBS buffer, pH 7.4), the cells were incubated with Abs against Myc or p65 for 1 h and then with a fluorophore-conjugated secondary Ab for an additional 1 h. The cell nuclei were stained with 4′,6′-diamidino-2-phenylindole (DAPI) for 5 min. The coverslips were sealed onto the glass slides using nail polish, and the slides were then subjected to fluorescent microscopy analysis. The images were taken with a Zeiss Axiovert 25 fluorescent microscope.

### Two-step Co-IP

HEK293T cells were co-transfected with HA-, MYC- and FLAG-Tagged proteins. Then, 36 h after transfection, the cells were lysed with lysis buffer, sonicated briefly and centrifuged. The supernatant was incubated with an anti-FLAG antibody bound to protein A/G-agarose beads for 3 h at 4 °C. The beads were washed three times with lysis buffer, and the FLAG-NMI protein complex was eluted with 300 μL of lysis buffer containing 250 mM NaCl and FLAG peptide for 3 h at 4 °C. The second immunoprecipitation was performed using 150 μL of the eluate and 350 μL of lysis buffer containing 300 mM NaCl and 5 μL of anti-HA antibody, followed by the addition of protein A/G-agarose beads.

### Cellular fractionation

Cells were washed twice with ice-cold PBS (pH 7.4) and resuspended in buffer A containing 10 mM HEPES (pH 7.9), 10 mM KCl, 1.5 mM MgCl_2_, 0.5 mM dithiothreitol (DTT), 1 mM PMSF. Cells were incubated on ice for 10 min, and then 0.5% final concentration NP-40 was added. Cell lysates were centrifuged at 15,000 × g for 15 min. The resulting supernatants were retained as the cytoplasmic fraction. The pellets were washed three times with buffer A and lysed in cell lysis buffer (20 mM Tris-HCl [pH7.5], 150 mM NaCl, 10 mM NaF, 20 mM β-glycerophosphate, 1 mM sodium orthovanadate, 1 mM PMSF, 10 μg/ml leupeptin, 2 μg/ml aprotinin, 1% Triton X-100, and 1 mM EDTA). The lysates were then centrifuged at 3000 × g for 10 min, and the supernatants containing nuclear proteins were recovered.

### Luciferase reporter assay

HEK293T, H1299 or U-2 OS cells were transfected in 6-well plates at 80% confluence with 0.5 μg different reporters, together with other plasmids in different combinations as indicated. Each sample was supplemented with 0.5 μg pCMV5-*LacZ*, which expresses β-galactosidase, for monitoring the transfection efficiency. Cells were collected and luciferase activity was measured at 24 h after transfection. All transfections were carried out in triplicate for at least three times, and error bars represent ± SD of the means.

### Statistical analysis

The significance of the differences between the group means was determined by Student’s t test. *p* < 0.05 was considered statistically significant.

## Electronic supplementary material

Supplementary Information
