# Supplementary material for: N-Myc-Interacting Protein Negatively Regulates TNF-α-Induced NF-κB Transcriptional Activity by Sequestering NF-κB/p65 in the Cytoplasm
Source: Sci Rep. 2017 Nov 6;7:14579. doi: 10.1038/s41598-017-15074-5 (PMC5674077; doi:10.1038/s41598-017-15074-5)
Supplement: Supplementary file 1 — Supplementary Information [file 41598_2017_15074_MOESM1_ESM.pdf]

N-Myc-Interacting Protein Negatively Regulates TNF-α-Induced NF-κB Transcriptional Activity by Sequestering NF-κB/p65 in the Cytoplasm

Jingjing Hou[1,2,3], Shihao Jiang[1], Jiabao Zhao[1] , Dong Zhu[1], Xinmeng Zhao[1], Jian-chun Cai[2, 3] and Si Qing Zhang[1, *]

[1]State Key Laboratory of Cellular Stress Biology and School of Life Sciences, Xiamen University, Xiamen, Fujian 361102, China; [2]Institute of Gastrointestinal Oncology, Medical College of Xiamen University, Xiamen, Fujian 361004, China; and [3]Department of Gastrointestinal Surgery, Zhongshan Hospital of Xiamen University, Xiamen, Fujian 361004, China

*Correspondence and requests for materials should be addressed to Si Qing Zhang (email: zhangsq@xmu.edu.cn).

Supplementary Figure 1

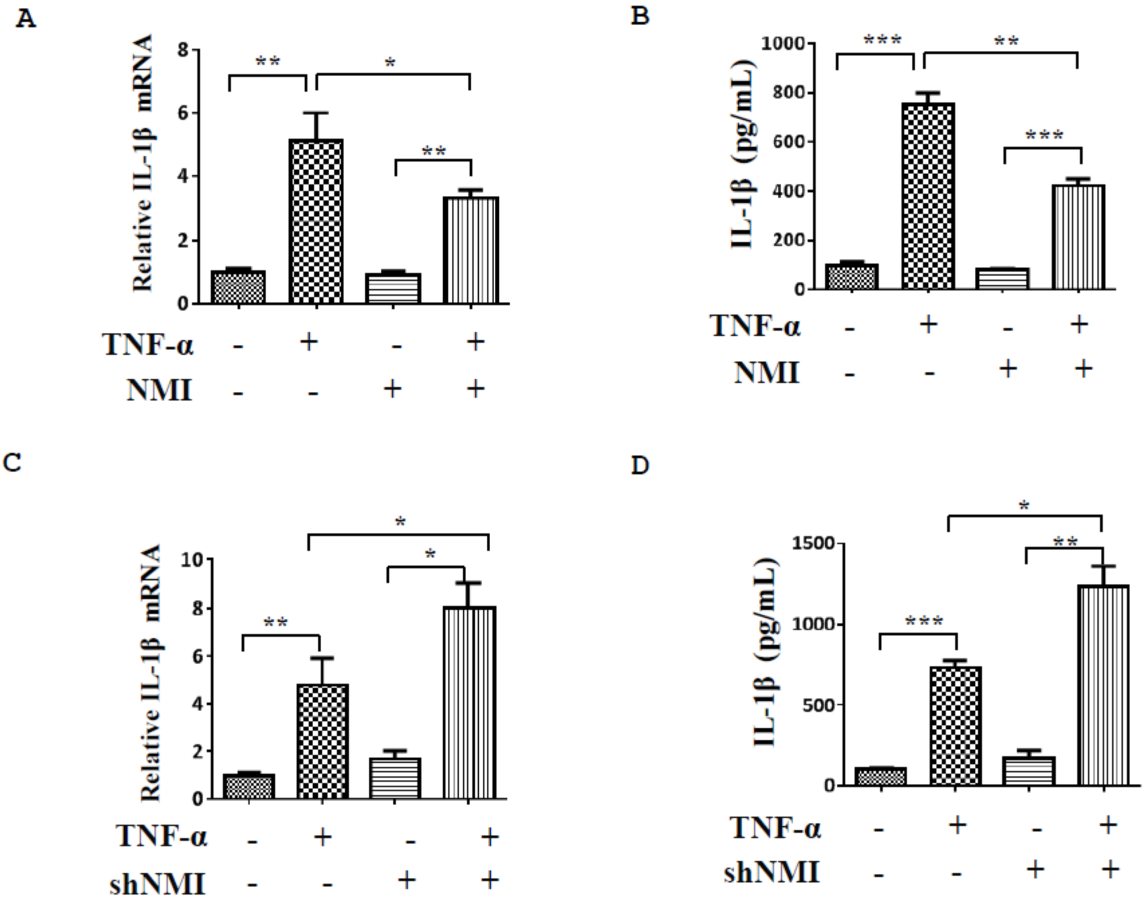

Supplementary Figure 2

A

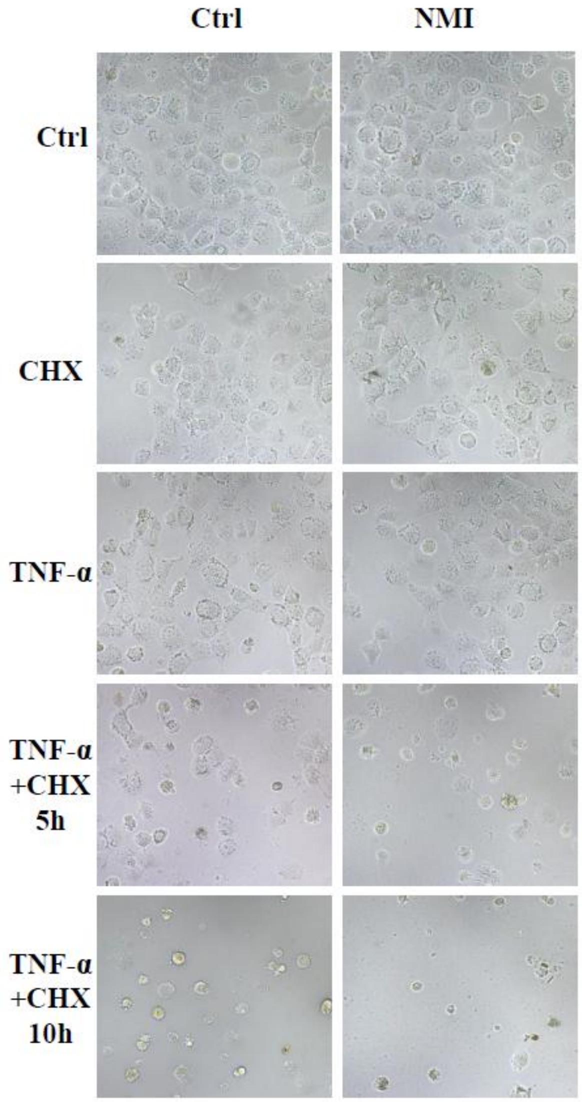

C

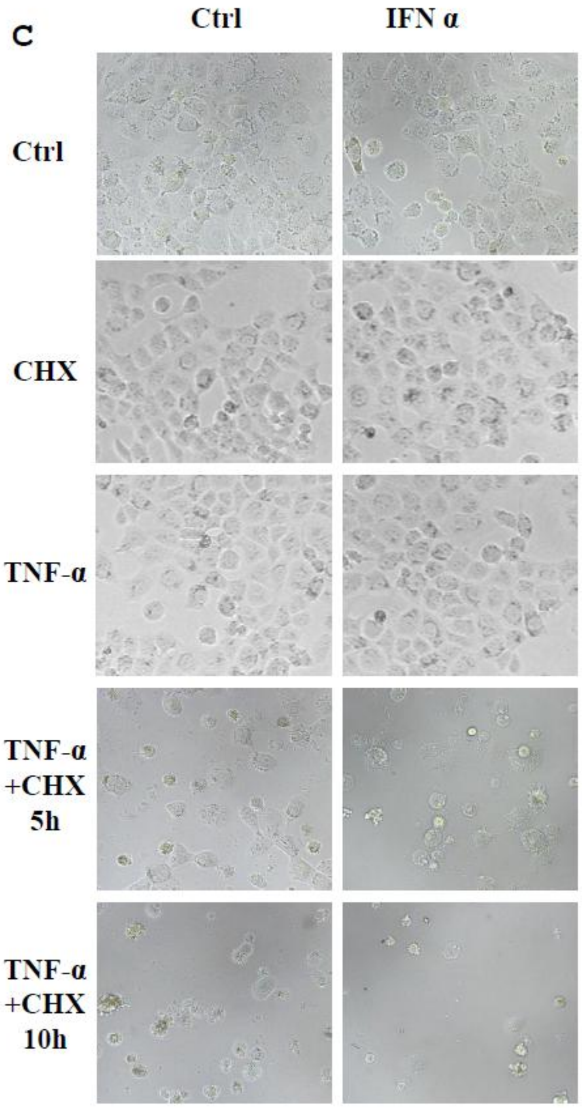

B

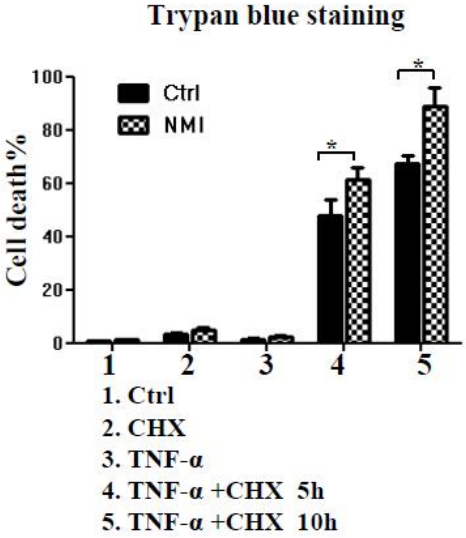

D

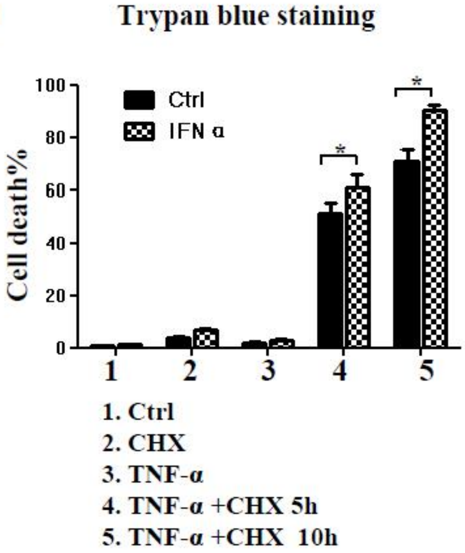

Supplementary Figure 3

A

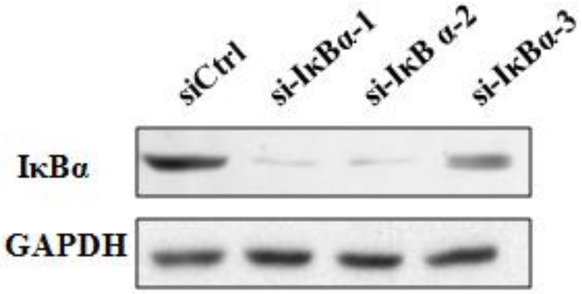

B

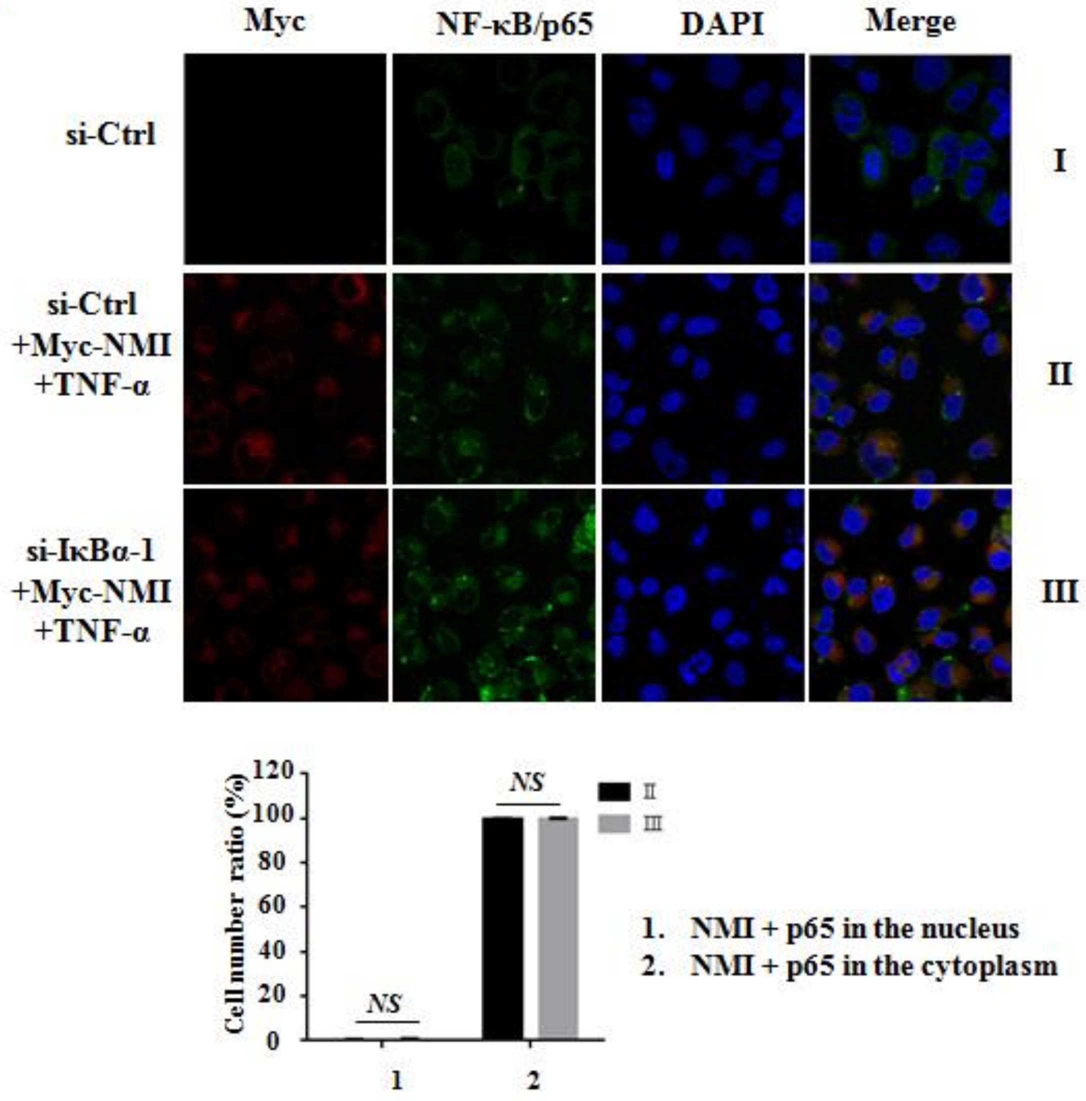

Supplementary Figure 4

A

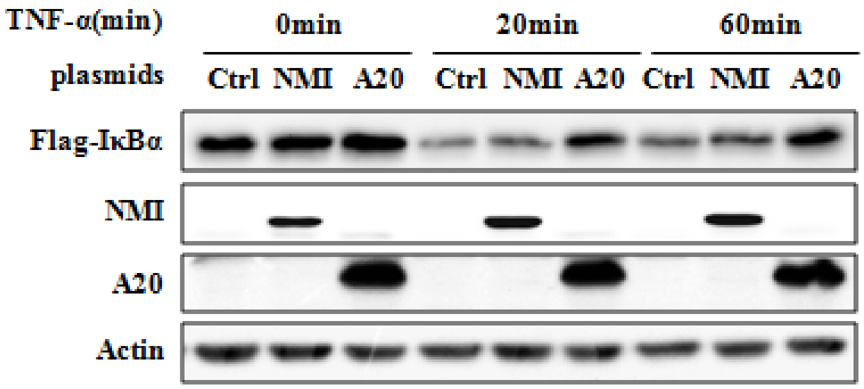

B

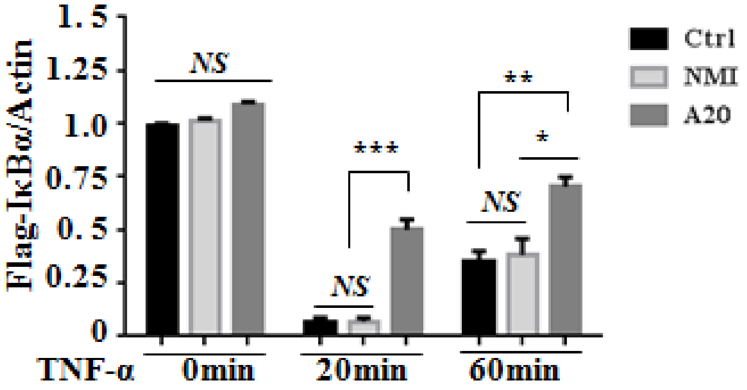

Supplementary Figure Legends

Supplementary Figure 1. NMI negatively regulates TNF-α-induced IL-1β production. **(A)** HeLa cells were transfected with control or NMI-expression plasmids, the cells were left untreated or treated with TNF-α (10 ng/ml) for an additional 2 h. The total RNA samples isolated from these cells were subjected to qPCR analysis using IL-1β, NMI, or GAPDH primers. **(B)** HeLa cells were transfected with control or NMI-expression plasmids, and the cells were left untreated or treated with TNF-α (10 ng/ml) for an additional 12 h. The IL-1β levels in the cell culture supernatants were assayed by ELISA. Results are representative of three independent experiments, and the error bars represent the SD. *$p < 0.05$, ***$p < 0.001$. **(C)** HeLa shCtrl or HeLa shNMI cells were left untreated or treated with TNF-α (10 ng/ml) for an additional 2 h. The total RNA samples isolated from these cells were subjected to qPCR analysis using IL-1β, NMI, or GAPDH primers. （**D**） HeLa-shCtrl and HeLa-shNMI cell lines were left untreated or treated with TNF-α (10 ng/ml) for an additional 12 h. The IL-6 levels in the cell culture supernatants were assayed by ELISA. Results are representative of three independent experiments, and the error bars represent the SD.*$p < 0.05$, **$p < 0.01$, ***$p < 0.001$.

Supplementary Figure 2. NMI enhances TNF-α plus CHX induced H1299 cell apoptosis.

(**A**) The NMI-overexpressing H1299 cells (H1299-NMI) show an increased apoptotic morphology after treatment with TNF-α plus CHX compared with the control cells

(H1299-Ctrl). H1299-Ctrl and H1299-NMI cells were plated in 12-well dishes. The following day, the cells were treated as indicated (TNF-α: 5 ng/ml, CHX: 5 μg/ml) for 5 h or 10 h, and the cells images were then taken with a Nikon-TE2000 microscope. (B) The cell death in (A) was quantified by a Trypan blue staining assay. The data are the mean ± S.D. of three independent experiments. $*p < 0.05$. **(C)** IFNα increases the apoptosis induced by TNF-α plus CHX in H1299 cells. H1299 cells were plated in 12-well plates, and the next day the cells were untreated or treated with IFNα for 12 h and then treated with TNF-α plus CHX for the indicated times (TNF-α: 5 ng/ml, CHX: 5 μg/ml). **(D)** The ratio of cell death in (C) was determined by a Trypan blue staining assay. The results are representative of three independent experiments, and the error bars represent the SD.$*p < 0.05$.

Supplementary Figure 3. HeLa cells were transfected with si-Ctrl or si-IκBα, 48h posttransfection, cells were transfected with Myc-NMI plasmid, and 24 h later, the cells were left untreated or treated with TNF-α (10 ng/ml) for 60 min. The cells were fixed and stained with anti-p65 or anti-Myc Abs and then with rhodamine-conjugated anti-mouse IgG (red) or FITC-conjugated anti-rabbit Abs (green). The same slide was also stained with DAPI for nuclear staining. Then, the expression and localization of p65 and Myc-NMI were determined by confocal immunofluorescence analysis. The percentage of the cells expressing p65 in the nucleus or cytoplasm among the cells that infected with si-Ctrl or si-IκBα was calculated in the bottom panel. *NS,* not significant.

Supplementary Figure 4. (A) HeLa cells were transfected with the FLAG- IκBα and control, Myc-NMI or A20 plasmids. At 24 h posttransfection, the cells were treated with TNF-α (10 ng/ml) for the indicated periods. The cell lysates were immunoblotted with the indicated Abs. **(B)** The quantification by densitometry of western blots of FLAG- IκBα bands was shown. Actin was used as the internal control. The results are representative of three independent experiments, and the error bars represent the SD. *$p < 0.05$, **$p < 0.01$, ***$p < 0.001$.
